# Perspectives: using polymer modeling to understand the formation and function of nuclear compartments

**DOI:** 10.1007/s10577-016-9548-2

**Published:** 2017-01-14

**Authors:** N. Haddad, D. Jost, C. Vaillant

**Affiliations:** 1 CNRS, Laboratoire de Physique, University of Lyon, ENS de Lyon, University of Claude Bernard, 69007 Lyon, France; 2 University Grenoble-Alpes, CNRS, TIMC-IMAG lab, UMR 5525, Grenoble, France

**Keywords:** Nuclear organization, genome, epigenome, copolymer model

## Abstract

Compartmentalization is a ubiquitous feature of cellular function. In the nucleus, early observations revealed a non-random spatial organization of the genome with a large-scale segregation between transcriptionally active—euchromatin—and silenced—heterochromatin—parts of the genome. Recent advances in genome-wide mapping and imaging techniques have strikingly improved the resolution at which nuclear genome folding can be analyzed and have revealed a multiscale spatial compartmentalization with increasing evidences that such compartment may indeed result from and participate to genome function. Understanding the underlying mechanisms of genome folding and in particular the link to gene regulation requires a cross-disciplinary approach that combines the new high-resolution techniques with computational modeling of chromatin and chromosomes. In this perspective article, we first present how the copolymer theoretical framework can account for the genome compartmentalization. We then suggest, in a second part, that compartments may act as a “nanoreactor,” increasing the robustness of either activation or repression by enhancing the local concentration of regulators. We conclude with the need to develop a new framework, namely the “living chromatin” model that will allow to explicitly investigate the coupling between spatial compartmentalization and gene regulation.

## Introduction: a multiscale compartmentalization of the genome

The compartmentalization of the genome is a clear hallmark of eukaryotic nuclear organization. In its pioneering work, almost a century ago, Emil Heitz introduced the terms of “euchromatin” and “heterochromatin” to account for the observed large-scale spatial density fluctuations of nucleus composition during interphase: as opposed to euchromatin, heterochromatin was referred to the chromosome “material” that remains densely stained during interphase (Brown 1966; Frenster et al. 1963; Pueschel et al. 2016). Further progresses in microscopy and immuno-staining/labeling techniques confirmed that euchromatic and heterochromatic compartments correspond actually to the aggregation of specialized functional chromatin: euchromatin is gene rich, displays higher expression level, and is generally more accessible and enriched for histone marks specific for active genes. In contrast, heterochromatin is more densely packed and harbors less genes and more repressive histone marks (Allis et al. 2007; Grewal and Jia 2007). Heterochromatin is usually classified into two subtypes: constitutive and facultative heterochromatin. Constitutive heterochromatin contains highly repetitive DNA sequences such as those found at (peri)centromeres and (sub)telomeres and serves to stably silence transposable elements (hence maintaining genome integrity). Facultative heterochromatin is typically associated with developmentally regulated genes whose chromatin structure may change in response to cellular differentiation signals. In many eukaryotes, from yeasts to plants and mammals, statistical analysis of hundreds of chromatin markers have identified only a small number of main chromatin types (Filion et al. 2010; Roudier et al. 2011; Julienne et al. 2013; Ho et al. 2014), typically 4 or 5, covering the well-known H3K9me2,3/HP1-like (SIR in budding yeast) constitutive heterochromatin or the facultative Polycomb-like heterochromatin but also a less-characterized ultra-repressive heterochromatin, the so-called black or null chromatin, enriched in genes that are expressed in few tissues (Filion et al. 2010; Julienne et al. 2013; Ho et al. 2014).

Since the early studies of nuclear organization using standard or electron microscopy, it is clear that heterochromatin and euchromatin occupy different compartments: repressed genes predominantly colocalize at the nuclear periphery, around the nucleolus and at the nuclear membrane, while the interior of nucleoplasm being rather transcriptionally active (Meister et al. 2011). Developmental cell specification is globally accompanied by a progressive spatial segregation of chromatin: starting from open and permissive chromatin organization in pluripotent ES cells to increasingly repressive, compact, and segregated state in differentiated cells (Meister et al. 2011; Zhu et al. 2013; Ahmed et al. 2010). Such spatial localization is in part mediated by interaction of heterochromatin with lamin proteins that form the nuclear lamina meshwork at the inner nuclear membrane (Kind and van Steensel 2014). Deregulation of such interactions during specific differentiation pathways may lead to global modification of the spatial chromatin organization (Solovei et al. 2013; Chandra et al. 2012, 2015).

**Fig. 1:**
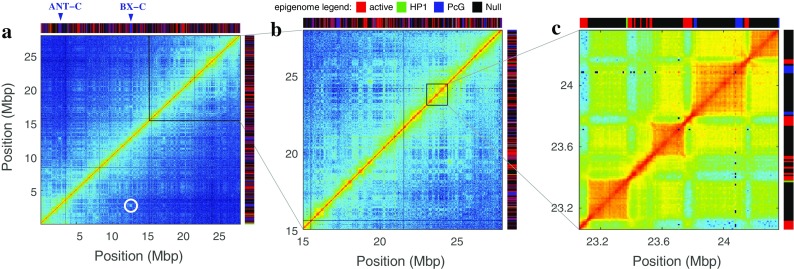
Compartmentalization of the genome as revealed by Hi-C maps. **a**–**c** Different magnifications of the Hi-C contact map of chromosome 3R performed in late embryonic cellular stages of *D. melanogaster* (Sexton et al. 2012) As clearly shown in **c**, the so-called TADs correspond to the higher self-interacting genomic domains and strikingly coincide with the active and inactive epigenomic domains obtained by Filion et al. (2010) (*colored-bar segmentation* on the top and right in **a**, **b**, and **c**) with (*red*): active chromatin, (*green*) HP1/H3K9me2–3 heterochromatin, (*blue*) PcG heterochromatin; and (*black*) null heterochromatin. Additionally, these maps reveal a typical checkerboard pattern corresponding to long-range (up to ~15Mbp) interactions between TADs of the same functional and chromatin state. In **a**, the white circle indicates the 10-Mbp long-range contact between the ANT-C and BX-C Polycomb domains

The recent application of next-generation sequencing techniques to map chromatin states (Chip-seq, RNA-seq, DamID, etc.) and pair-wise contacts (chromosome conformation capture methods) (de Wit and de Laat 2012) at the genome-wide level, as well as the development of super-resolution microscopy (Boettiger et al. 2016; Fabre et al. 2015; Wani et al. 2016), have essentially provided a more refined view of the link between genome compartmentalization and activity. Hi-C and 5C experiments revealed that chromosomes are segmented into kbp- to Mbp-long contact domains (Nora et al. 2012; Dixon et al. 2012; Sexton et al. 2012), the so-called topologically associating domains (TADs) (Fig. 1a, b). TADs define genomic regions with higher propensities to self-contact, accompanied with partial contact insulation with neighboring TADs (Dixon et al. 2012) (Fig. 1b), thereby segmenting the 1D genome into 3D domains. This has been also recently confirmed by immuno-labeling and super resolution microscopy (Boettiger et al. 2016; Fabre et al. 2015; Wani et al. 2016; Wang et al. 2016). The presence of TADs or similar 3D domains has been documented in most species in which genome-wide 3C has been carried out ranging from bacteria and yeasts to mammalian cells and plants (Dekker and Heard 2015). Most effective promoter-enhancer interactions occur inside the same TAD (Dixon et al. 2012; Ghavi-Helm et al. 2014), suggesting that the TAD environment promotes regulatory interactions while sufficiently preventing putatively deleterious interactions between promoters and enhancers located in neighboring TADs. Indeed, deletion of a TAD boundary is sufficient to induce ectopic new contacts with regulatory sequences in the neighboring TAD and may lead to aberrant gene expression (Guo et al. 2015, Lupianez et al. 2015, Lupiáñez et al. 2016, Franke et al. 2016). The TAD structure of the genome is remarkably invariant over the course of development, between different cell types or even between species in conserved synteny blocks (Dixon et al. 2015; Lonfat et al. 2014; Jin et al. 2013; Le Dily et al. 2014). Experimental data revealed that the epigenomic composition of TADs is rather uniform in either active or inactive epigenetic marks (Rao et al. 2014; Sexton et al. 2012; Le Dily et al. 2014) (Fig. 1b) while TAD boundaries are enriched in a number of chromatin-binding proteins (like CTCF or cohesin) or in specific epigenomic states (Rao et al. 2014; Sexton et al. 2012; Dixon et al. 2012; Ho et al. 2014; Zhu et al. 2016; Fraser et al. 2015). As illustrated in Fig. 1b, there is a remarkable match between the spatial (3D) genomic compartmentalization into TADs and the linear (1D) segmentation into epigenomic domains: in *Drosophila* (and to a less extent in human), sequences from the same epigenomic domains tend to self-associate more frequently. At the megabase pair (Mbp) scale, contact maps display a cell type-specific checker board pattern (Rao et al. 2014; Lieberman-Aiden et al. 2009; Sexton et al. 2012) (Fig. 1a–c) enlightening a multiscale organization with a rather complex pattern of long-range contacts between TADs of the same chromatin state (Fig. 1b) (Rao et al. 2014; Lieberman-Aiden et al. 2009; Wang et al. 2016; Fraser et al. 2015).

Altogether, these experimental results suggest that the 1D heterogeneous epigenomic landscape is hierarchically organized into distinct 3D nuclear compartments. However, the molecular and physical mechanisms responsible for such multiscale organization are still unclear. In the recent years, polymer modeling of chromatin folding has emerged as a powerful tool to validate or suggest putative mechanisms and to get new insights into the formation of nuclear compartments. In particular, heteropolymer models that explicitly consider the coupling between chromatin structure and function have recently started to emerge (Barbieri et al. 2012; Brackley et al. 2016; Jerabek and Heermann 2012; Benedetti et al. 2014; Doyle et al. 2014; Ganai et al. 2014; Jost et al. 2014; Tark-Dame et al. 2014; Nazarov et al. 2015; Sanborn et al. 2015; Ulianov et al. 2016; Fudenberg et al. 2016; Tiana et al. 2016). In this perspective article, we are going to focus on some recent ideas (Jost et al. 2014; Olarte-Plata et al. 2016) concerning the connection between epigenome, polymer physics, and the formation of sub-Mbp domains (TADs) inside chromosome territories. Then, we will discuss the possibility that the genome compartmentalization might not be only the consequence of the genome function (the epigenome) but might actually serve as a “nanoreactor” enhancing robustness of local biochemical reaction involved in gene regulation and in particular in chromatin assembly and maintenance.

## From 1D to 3D: quantitative modeling of (epi-)genome folding

### Homopolymer modeling of “generic” folding properties

**Fig. 2:**
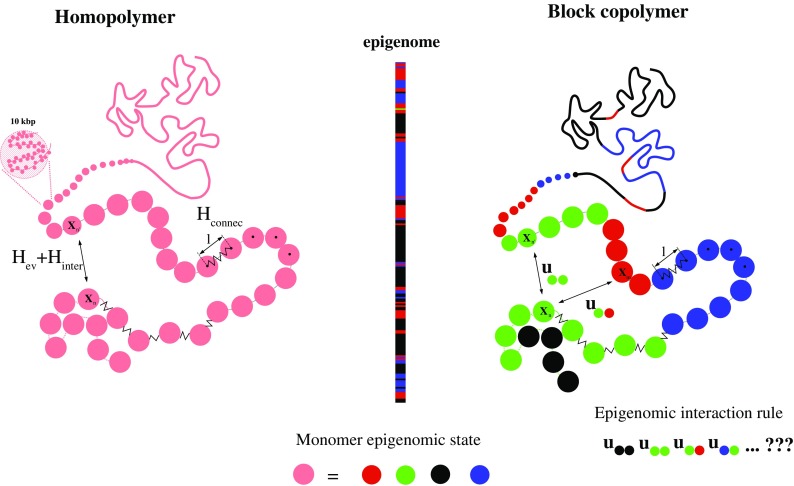
Coarse-grained bead-spring polymer models of chromatin (**left**) homopolymer model and **(right)** Block copolymer model. One block corresponds to one epigenomic domain. Monomer-monomer attractions depend on the local epigenomic state. In *Drosophila*, we consider four chromatin states (Filion et al. 2010): active (red), HP1/H3K9me2/3 heterochromatin (green), PcG heterochromatin (blue), and null heterochromatin (black)

**Fig. 3:**
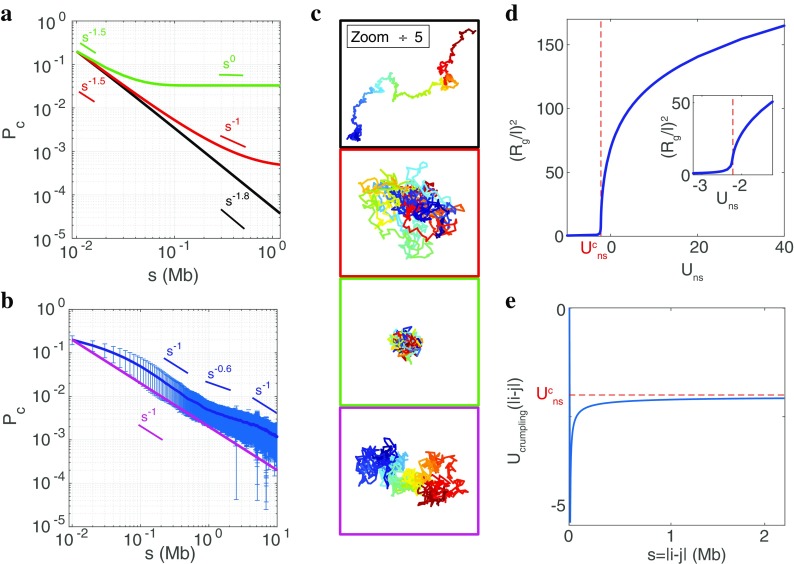
Homopolymer as a model for generic folding properties. **a** Theoretical *P*_*c*_(*s*) computed from the GSC approach when considering a homopolymer (*N* = 1000) with *U*_*ns*_ =  − 1.9 *k*_*B*_ *T* (black), ${U}_{ns}={U}_{ns}^c=-2.2{k}_BT$ (red), *U*_*ns*_ =  − 2.9*k*_*B*_ *T* (green). **b** Sequence-averaged experimental contact probability computed from Hi-C experiments performed at late *Drosophila* embryonic stage (Sexton et al. 2012) (*dark blue*); fluctuations are represented by the *error bars* (*light blue*). Theoretical *P*_*c*_(*s*) for the homopolymer model with the «crumpling» non-specific interaction potential *U*_*crumpling*_(*i*, *j*) (see text) and *U*_*ns*_ = 0 (magenta). **c** Typical configurations associated to the models reported in **a** and **b**, same color code. **d** ${R}_g^2/{l}^2$vs *U*_*ns*_, where ${R}_g=\sqrt{1/\left(2{N}^2\right)\sum {D}_{ij}}$ is the radius of gyration. The *vertical dashed line* indicates the collapse transition at ${U}_{ns}^c=-2.2{k}_BT$. *Inset*: zoom around the collapse transition. **e** Crumpling potential: *U*_*crumpling*_(*i*, *j*) = *U*_*crumpling*_(*s* = |*j* − *i*|)

Several models based on polymer physics were suggested in the last years, providing an interesting starting point to understand the minimal requirements for the creation of higher-order chromatin structures. Before the development of a more elaborated model, it is instructive to consider chromatin as a simple homogeneous polymer (Fig. 2 left). This is obviously not the case, but such null model may already provide fundamental insights concerning the generic feature of higher-order chromatin organization. By “generic,” we mean the sequence-averaged conformational properties such as the average evolution of the contact frequencies *P*_*c*_ (Fig. 3b) or distance between two loci as a function of their distance *s* in base pairs along the linear genome. In such homogeneous models, chromatin is described by a chain of connected identical monomers (modeled by beads of diameter l) whose dynamics is controlled by thermal forces and excluded volume interactions (monomers cannot overlap) and eventually by the rigidity of the chromatin fiber or by non-specific interactions between monomers.

More precisely, we consider a chain (a chromosome or a piece of chromosome) with N monomers, each monomer representing 10 kbp. Any spatial configuration is fully characterized by the coordinates *X* = {*X*_*i*_ = (*x*_*i*_, *y*_*i*_, *z*_*i*_)}_*i* = 1 .  .  . *N*_ of the beads. The energy associated to any conformation is given by the Hamiltonian of the system, which can be written in a very general form as *H* = *H*_*chain*_ + *H*_*interaction*_. The first term *H*_*chain*_ = *H*_*connectivity*_ + *H*_*ev*_ accounts for chain connectivity and excluded volume: (i) ${H}_{connectivity}=k/2\sum {r}_{i,i+1}^2$ with ${r}_{ij}^2={\left({X}_i-{X}_j\right)}^2$and *k* = 3*k*_*B*_ *T*/*l* ^2^, (ii) the excluded volume interactions are modeled by Gaussian repulsive potentials: ${H}_{ve}=\sum {U}_{ev}\mathit{\exp}\left(-{r}_{ij}^2/2{r}_e^2\right)$, with *U*_*ev*_ = 10*k*_*B*_ *T* and *r*_*e*_ = 0.3*l*. The second term *H*_*interaction*_ accounts for the non-specific interactions between monomers: ${H}_{interaction}=\sum {U}_{ns}\mathit{\exp}\left(-{r}_{ij}^2/\left(2{r}_o^2\right)\right)$, with *r*_*o*_ = 0.5*l* and *U*_*ns*_ a non-specific short-range interaction term.

There are different ways of investigating such model: standard molecular dynamics (Rosa and Everaers 2008) or kinetic Monte-Carlo simulations (Olarte-Plata et al. 2016), Gaussian self-consistent (GSC) approximation (Ramalho et al. 2013; Jost et al. 2014), etc. The qualitative behavior of the system is independent of the chosen method. In the following, we will exclusively present results based on the GSC approach that has been described in details by us in Jost et al. (2014). Briefly, the idea is to approximate at each time-step the “true” distribution of probability *P*(*X* = {*Xi*}, *t*) for a given conformation *X* by a multivariate Gaussian distribution. Such distributions are fully characterized by the covariance matrix *C* or equivalently by the squared distance matrix *D* with *D*_*ij*_ = 1/3〈(*X*_*i*_ − *X*_*j*_)^2^〉 = *C*_*ii*_ + *C*_*jj*_ − 2*C*_*ij*_ (where ⟨•⟩ stands for average over the distribution P). This approximation leads to a self-consistent equation for *D* at steady state: 0 = 4*k*_*B*_ *T* −  ∑ (*J*_*ik*_ − *J*_*jk*_)(*D*_*ik*_ − *D*_*jk*_), where *J* is a non-linear function of *D* and of the model parameters. This equation can be efficiently solved by iterative methods (Haddad 2016). Then, from every steady-state distance matrix *D*, it is possible to derive all the statistical properties of the chain (contact map, radius of gyration *Rg* etc.) as well as generate representative 3D configurations.

As shown in Fig. 3, the GSC approach applied to the classical homopolymer model faithfully recovers the basic folding features of a single chain: (1) for non-specific repulsion (*U*_*ns*_ > 0) and weak attraction, the chain behaves as a self-avoiding walk with *P*_*c*_ ∝ *s* ^−2^ and extended coil configurations (black in Fig. 3a, c); (2) when increasing the non-specific attraction *U*_*ns*_ (as a proxy for modeling the effect of a confinement for example), there is a critical value ${U}_{ns}^c\sim -2.2{k}_BT$ where the chain experiments a collapse transition as revealed by the drop of the radius of gyration (Fig. 3d) toward a compact globular state for ${U}_{ns}<{U}_{ns}^c$, characterized by a constant contact probability above a typical small distance (green in Fig. 3a, c). At the transition (red in Fig. 3a, c), the chain is Gaussian and *P*_*c*_(*s*) ∝ *s* ^−3/2^ over a large range. Such critical regime provides a rather good description of the generic properties of small genomes like budding yeast (Wong et al. 2012; Kimura et al. 2013; Avşaroğlu et al. 2014).

In higher organisms such as flies, humans, or mice, having long chromosomes and large-scale organization is characterized by contact frequencies that evolve as *P*_*c*_(*s*) ∝ *s* ^−1^(Fig. 3b) for (100*kbp* < *s* < 10 *Mbp* for flies, 500 *kbp* < *s* < 5 *Mbp* for mammals) and by the existence of chromosome territories, i.e., by weak chromosome intermingling (Lieberman-Aiden et al. 2009; Dixon et al. 2012; Sexton et al. 2012). It has been shown that such features are compatible with unknotted self-similar polymeric conformations, the so-called fractal or crumpled globule models (Lieberman-Aiden et al. 2009; Grosberg et al. 1993; Mirny 2011), which are likely to result from the out-of-equilibrium folding of long polymers at high volumic density (Rosa and Everaers 2008; Bohn and Heermann 2010; Halverson et al. 2014; Gürsoy et al. 2014). Due to chain non-crossability, the relaxation time to the equilibrium scales as *N* ^3^ such that long chromosomes retain the partial memory of the initial postmitotic (putatively knot-free) state for a very long time, often much longer than a typical cell cycle. For example in *Drosophila*, a series of FISH experiments performed at different developmental stages clearly revealed a long persistence of the initial Rabl-like postmitotic organization with a progressive loss after several hours (Dernburg et al. 1996; Csink and Henikoff 1998; Lowenstein et al. 2004; Harmon and Sedat 2005).

Within the GSC approximation, it is not possible to account for the crumpling of chromosome with the simple model introduced above. In order to account for this generic behavior of long chromosome, we introduce a non-specific effective pairwise attractive contribution to ${H}_{inter}=\sum {U}_{crumpling}\left(i,j\right)\mathit{\exp}\left(-{r}_{ij}^2/\left(2{r}_o^2\right)\right)$_._ *U*_*crumpling*_(*i*, *j*) only depends on the genomic distance *s* = |*j* − *i*| (Fig. 3e) and was fitted to exactly provide in our GSC framework a stationary state with *P*(*s*) ∝ *s* ^−1^ in the absence of other non-specific contributions (*U*_*ns*_ = 0) (magenta in Fig. 3b, c). Interestingly, for large *s*values, *U*_*crumpling*_(*s*) is very close to the critical strength of the non-specific interaction (${U}_{ns}^c$) where the collapse transition occurs in the previously described homopolymer model (no crumpling, Fig. 3a, d). This means that the crumpling potential drives the system close to the collapse transition and that small perturbations around the crumpled state (by adding a non-zero *U*_*ns*_ term) may lead to coiled (*U*_*ns*_ > 0) or globular (*U*_*ns*_ < 0) configurations.

### Forming the compartments: the copolymer framework

Homopolymer models give a rather good description of the general large-scale organization of chromatin. However, they obviously fail to account for the multiscale compartmentalization into TADs or into higher hierarchies that are likely to depend on the local genomic or epigenomic composition. Here, we discuss how heteropolymer models accounting for such specificities may improve the description of chromatin folding.

Chromatin is now modeled as a block copolymer where blocks correspond to consecutive monomers with an identical chromatin state (Fig. 2 right). In addition to the non-specific monomer-monomer attraction *U*_*ns*_ and the crumpling potential *U*_*crumpling*_, we thus introduce a specific attraction term: ${H}_{interaction}=\sum {U}_s\left({e}_i,{e}_j\right)\mathit{\exp}\left(-{r}_{ij}^2/\left(2{r}_o^2\right)\right)$ in *H*_*inter*_ that depends on the chromatin states *e*_*i*_ and *e*_*i*_ of monomers *i* and *j*. To simplify, we assume that only monomers of the same state interact with each other and that the strength of interaction is the same for each state. As already noticed in Jost et al. (2014), such epigenomic-driven attractions reflect the ability of some proteins that compose the chromatin state to oligomerize and thus potentially bridge two distant genomic sites of the same chromatin state (Canzio et al. 2013, Isono et al. 2013, Hiragami-Hamada et al. 2016).

**Fig. 4:**
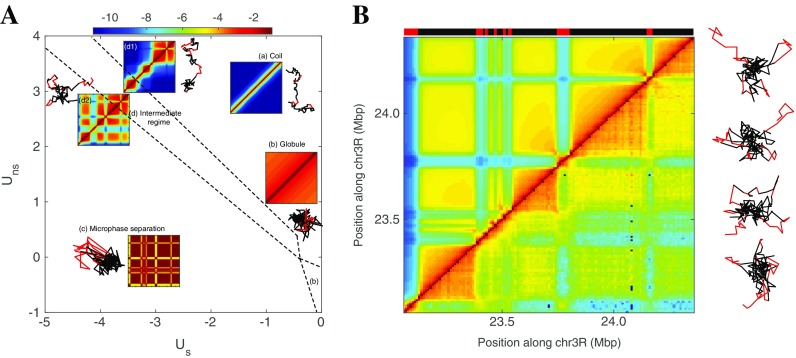
The phase diagram of the copolymer framework for modeling epigenome folding. **A** Phase diagram of the copolymer representing the region located between position 23.06 and 24.36 Mbp of the chromosome 3R as a function of the strength of non-specific *U*_*ns*_ and specific *U*_*s*_ interaction (in *k*_*B*_ *T* units) when considering the crumpling interaction term. Blocks of the copolymer are built from the (simplified) two-state epigenomic segmentation reported in **B** and original segmentation and experimental contact map associated to this fragment are reported in Fig. 1c. The phase diagram is obtained by computing the stationary states derived from the GSC approach. *Dashed lines* represent the resulting continuous transitions between the different phases computed by monitoring the radius of gyration of the whole chain, of single epigenomic domain, or of all the monomers of the same state as a function of parameters (see Jost et al. 2014; Haddad 2016). The grid pitch is 0.2 *k*_*B*_ *T*. *Insets* represent typical structures and heat maps of the probability of contacts between two monomers (in the same log-unit color-scale) for the different phases: *(a)* coil (*U*_*ns*_ = 3; *U*_*s*_ =  − 1), *(b)* globule (*U*_*ns*_ =  − 0.4; *U*_*s*_ = 0), *(c)* multiphase separation (*U*_*ns*_ = 1; *U*_*s*_ =  − 3.4), and *(d)* intermediate regime (*U*_*ns*_ = 3; *U*_*s*_ =  − 3.4). **B** Comparison between the experimental contact map (*right bottom*) and the best prediction (*top left*) obtained with *U*_*ns*_ = 2 and *U*_*s*_ =  − 2.6; typical configurations associated with this predicted state are reported on the *right*

As an illustration of the copolymer framework with the GSC approach, we consider a genomic region of chromosome 3R whose contact map and epigenomic segmentation are reported in Fig. 1c. This region is mainly composed of two types of epigenomic domains: active (“red” chromatin) and inactive (“black” chromatin). By varying the strength of non-specific and specific interactions, the systems exhibit a variety of different phases (Fig. 4A) (Jost et al. 2014; Olarte-Plata et al. 2016). For weak interactions, configurations are characteristic of an unstructured, coil phase. For strong attractive interactions, a microphase separation is observed and large portions of monomers of the same state occupied separate spatial compartments leading to strong checkerboard patterns (Fig. 4A, B). In the intermediate regime, the systems show a continuous crossover between the coil and the microphase regimes. We observe the partial internal collapse of blocks into TAD-like domains, followed by the appearance of weak long-range stochastic interactions between TADs of the same chromatin state. The corresponding 3D compartments may contain several TADs but are transient and only weakly collapsed. As the interactions become more attractive, the blocks experience an internal theta-collapse transition to an equilibrium globule and long-range interactions become more and more important, leading to the formation of long-lived larger 3D compartments. The precise shapes of the phase diagram, as well as the behavior of individual blocks, are strongly dependent on the underlying pattern of chromatin states (size of blocks, number of different states, etc.) (Jost et al. 2014; Olarte-Plata et al. 2016). For example, larger blocks will start collapsing at weaker interaction strength due to stronger collective effects (Olarte-Plata et al. 2016).

As shown in Fig. 4B, experimental HiC data are compatible with the intermediate regime where chromatin blocks have partially collapsed into TADs and where blocks of the same state transiently merge together into dynamic 3D compartments resulting in the characteristic weak checkerboard pattern observed in HiC maps. This observation is consistent with FISH microscopy experiments of Polycomb bodies, spatial compartments associated with facultative heterochromatin, showing that such bodies are indeed highly dynamic inside the fly nucleus (Cheutin and Cavalli 2012) but also in human (Vieux-Rochas et al. 2015). In this intermediate regime, prediction of the time-evolution of the contact maps shows that TADs form quickly first, followed by the slow formation of long-range interaction (Jost et al. 2014). This is again in agreement with HiC data on synchronized cells along the cell cycle (Naumova et al. 2013). Another property of systems in this regime is the internal compaction of TADs that increases with the TAD size for a given interaction strength. In *Drosophila*, this simple prediction agrees nicely with the measurements on heterochromatic TADs (Olarte-Plata et al. 2016; Boettiger et al. 2016). Interestingly, for active, euchromatic domains, the compaction does not depend on the size, which again point out that active chromatin only weakly interacts with itself. This may reflect a distinct local mode of interaction between chromatin types: active chromatin rather organizes locally via pairwise short-range bridging between discrete specific genomic sites while heterochromatin may interact more continuously via clustering of multiple chromatin loci. This is consistent with more homogeneous internal contact patterns observed for inactive domain and more complex interactome profiles for active domains (Sofueva et al. 2013).

It is interesting to note that the intermediate regime—compatible with experiments—arises from finite-size effect (Care et al. 2015; Cortini et al. 2016) and should not be observed for long block copolymer at equilibrium. Is it paradoxical, knowing that chromosomes are actually very long polymers? Here, we have to remind us one of the main lessons from Rosa and Everaers (2008): HiC data at large scales are compatible with the out-of-equilibrium decondensation of a topologically constrained long polymer. This suggests that partial equilibrium or stationary state is only achieved locally and that at large scale, dynamics is so slow that only few configurations might be explored, possibly leading to finite-size effects at smaller scale. Hence, local equilibrium finite-size effects might emerge from the out-of-equilibrium slow dynamics of a very long polymer.

Recent works have shown that chromatin folding in higher vertebrates like mammals can also be well described by copolymer models using genomic/epigenomic contact interactions (Brackley et al. 2016; Chiariello et al. 2016; Di Pierro et al. 2016). However, the observation that strong loops detected between TAD boundaries occur mainly between convergent CTCF sites (Dowen et al. 2014, Rao et al. 2014; Vietri Rudan et al. 2015) is incompatible with TAD formation mechanisms based only on short-range interactions (Fudenberg et al. 2016; Sanborn et al. 2015). To account for that, it was recently proposed that TAD formation in mammals might be driven by a loop extrusion mechanism (Fudenberg et al. 2016; Sanborn et al. 2015): extruder factors, putatively cohesin rings, bind to chromatin and extrude sequentially large DNA loops until unbinding or pausing at CTCF-bound sites having the proper orientation. Development of polymer models combining loop extrusion and epigenomic-driven interactions would allow understanding more globally chromatin folding in mammals from TAD formation to inter-TAD long-range interactions.

### Toward a predictive model: GSC-based inference of epigenome-specific interaction parameters

The copolymer framework associated with the self-consistent Gaussian approximation may represent an efficient formalism to extract from the available experimental data the effective genomic and epigenomic interactions between chromatin loci. A promising outcome of such inference process would be a powerful model able to predict the chromatin organization in various conditions, allowing investigating in silico changes in TAD formations and long-range contacts when altering the epigenome. This may provide a very interesting framework for understanding how modifications of the epigenome during development or perturbations associated to diseases could lead to cell phenotypic variations via large-scale chromatin reorganization.

**Fig. 5:**
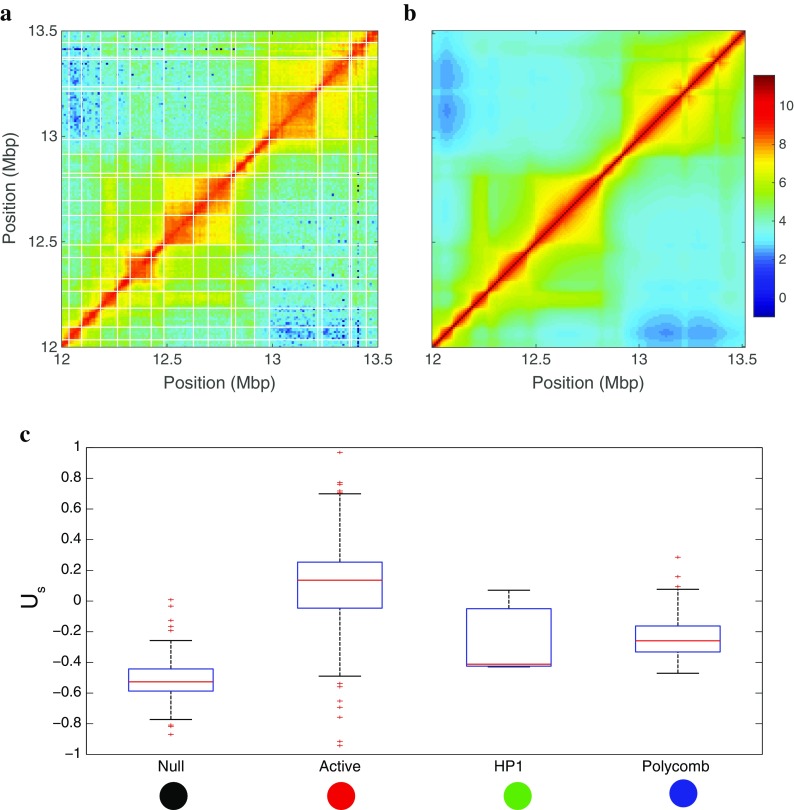
Inference of interaction parameters. **a** HiC-maps (1–2 Mbp) are first partitioned into TADs using IC-Finder (Haddad et al. 2017; http://membres-timc.imag.fr/Daniel.Jost/DJ-TIMC/Software.html). We assume that monomers within the same TAD interact similarly. Using GSC coupled to gradient-descent-like algorithm (Haddad 2016), we find the set of parameters (intra-TAD and inter-TAD interaction strength) that minimizes the gap (measured by a *χ* ^2^-score) between the prediction and the experimental data. **b** Example of a predicted map obtained after minimization; the target experimental map is the one shown in **a**. **c** Distribution of intra-TAD values obtained when applying the inference scheme to the whole *Drosophila* genome (on sliding windows of size 1–2 Mbp) as a function of the majority chromatin state in the TAD

For that purpose, we have developed a scheme that allows inferring the monomer-monomer-specific interactions matrix *U*_*s*_(*i*, *j*) that describes at best the experimental contact maps (Fig. 5 and legend). To reduce the number of parameters and to strengthen the robustness of the inference regarding the presence of strong uncertainties in the experimental data, we apply the method at the TAD level, assuming that monomers within the same TAD interact similarly (*U*_*s*_(*i*, *j*) = *U*_*s*_(*i*′, *j*′) if *i* and *i*′ are in the same TAD and idem for for *j* and *j*′). Figure 5b shows that this approximation leads anyway to a very good match with experimental map.

We applied the inference scheme to the whole *Drosophila* genome and asked to what extent the inferred specific attractions were dependent on the local chromatin states. In Fig. 5c, we plot the distribution of *U*_*s*_ values obtained for TADs as a function of their main epigenomic state. As expected, heterochromatic states (PcG, HP1, and null states) self-attract more (−0.2*kT* < *Us* <  − 0.6*kT*) than do active chromatin (*U*_*s*_ ≈ 0). This is again coherent with the observations that repressed genes are embedded in more compact environments.

These preliminary results pave the way to the development of quantitative and predictive descriptions of chromatin folding based on epigenomic information in higher eukaryotes. Recently, similar attempts to parameterize copolymer-like models based on human HiC-data (Giorgetti et al. 2014; Brackley et al. 2016; Chiariello et al. 2016; Di Pierro et al. 2016) have also led to quantitative and predictive descriptions of chromatin folding however showing a weaker association between epigenomic data and specific attractions.

## From 3D to 1D: spatial chromatin compartments and the nanoreactor hypothesis

### Functional importance of spatial compartments: increasing the local concentration

As discussed before, the spatial organization of chromatin results in part from the clustering of epigenomic chromatin states but a still open question is whether this spatial organization is only a byproduct of (epi-) genome activity or is also participating in the regulation of the epigenome assembly and more generally in the regulation of the genome function. The basic concept behind this structural/functional coupling is the increase of local concentration of regulatory proteins due to spatial colocalization. This paradigm has been actually evidenced and formalized for many years in the context of the well-known lac operon system (Oehler et al. 1994, 2006, Oehler and Müller-Hill 2010; Vilar and Leibler 2003). Molecular crowding and spatial confinement increase the binding affinities of regulators (activators and repressors) to their chromatin/DNA-targeted regulatory sequences (ibid). In some sense, the nuclear compartments would correspond to biochemical nanoreactors where a few number of reacting biomolecules are colocalized in space, favoring their biochemical (co)activity on chromatin and *in fine* on DNA.

In the lac system, the presence of few additional dispersed recruitment sequences (operators) and the ability of the lac-repressor to oligomerize and enhance the association of a repressor to the effective “repressing” site (Vilar and Leibler 2003). In eukaryotes, similar strategies are acting at the level of enhancer-promoter modules where the action at “distance” of the enhancer sequences are conditioned to their physical contacts with the promoter: as for the lac system, distal enhancer sequences might actually act as secondary recruitment sequences for TFs that, by associating with mediator and other architectural proteins, can promote recruitment and stabilization of the transcriptional machinery at promoters via long-range looping and clustering (Spitz 2016; Liu et al. 2014).

Along the same line, in *Drosophila*, PcG-mediated gene repression involves the spatial colocalization of the silencer elements PREs into 3D compartments, the so-called PcG bodies, mediated by the Polycomb proteins (Wani et al. 2016; Lanzuolo and Orlando 2007; Bantignies et al. 2011; Cheutin and Cavalli 2012). Such clustering operates “*in cis*,” i.e., within an epigenomic domains, but also “*in trans*” between distant domains as for example between the ANT-C and BX-C domains (white circle in Fig. 1a) where the level of repression has been directly correlated to the level of clustering between PcG domains (Bantignies et al. 2011). Same colocalization mechanisms of PcG-repressed genes are also observed in mammals (Vieux-Rochas et al. 2015). In budding yeast, repression by the SIR system has been linked to spatial clustering and perinuclear anchoring of SIR-bound telomeres at the nuclear membrane (Meister and Taddei 2013). And, a similar coupling between clustering and repression at the nuclear envelope also operates in higher eukaryotes such as worms and mammals (Meister and Taddei 2013), which has been recently remarkably evidenced in the chromosome-wide inactivation process of the X chromosome (Chen et al. 2016).

**Fig. 6:**
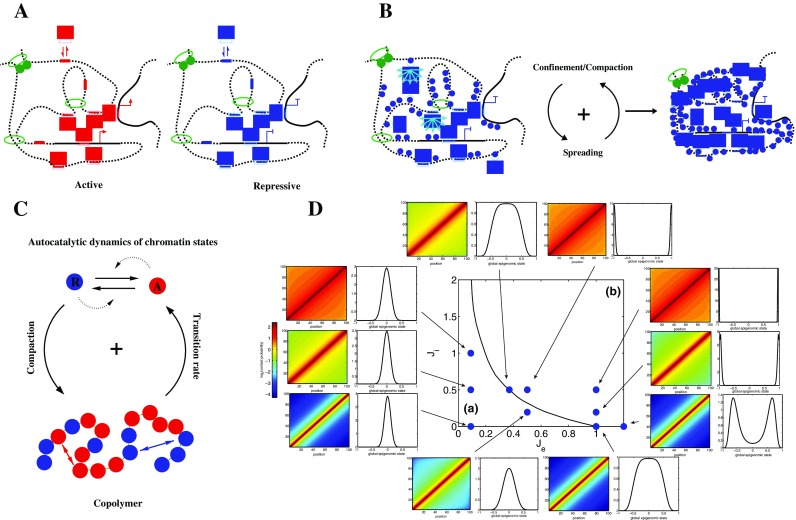
Self-assembly of nanoreactors and the living chromatin model. **A** The spatial confinement of genomic regulatory sequences such as enhancers (*red segments*) or silencers (*blue segments*) result in the increase of the “local” concentration of activators (*red rectangles*) or repressors (*blue rectangles*) that are targeted to these sequences; the formation of these active or repressive compartments can result from the self-association between the bound regulators and/or by independent mechanisms such as loop extrusion. **B** The ability of regulators to both associate with given chromatin markers (*blue dots*) and propagate these markers *in cis* and *in trans* (*light blue arrows*) provide a robust way of maintaining the functional compartments. **C** The living chromatin model is a model that combines the copolymer framework (self-attraction between monomer of same chromatin state) and the epigenome dynamics framework (autocatalytic conversion between states); here, as in **D**, we consider a simple case with two states: repressive (−1) (*blue*) or active (+1) (*red*). **D** Phase diagram of the living chromatin model for a small genomic region as function of the epigenomic-spreading strength *J*_*e*_ and of the specific interaction strength *J*_*i*_. The *black line* separates a region where no global epigenomic state can be defined (*a*) and a region where a coherent active or inactive state is stabilized (*b*). For several points of the diagram, we plot the predicted contact probability map (*right*) and the probability distribution of the global epigenomic state (*left*) defined as the mean value of *e*_*i*_ in the region (∑*e*_*i*_)/*N*

The mechanisms that drive this nanoreactor formation has been discussed before: the polymeric nature of chromatin induces a “natural” confinement since dispersed sequences on the same chain have a greater probability to colocalize due to chain looping. Every process that promotes this looping probability also enhances local confinement. In particular, the multimerization of regulatory DNA-binding proteins can promote physical bridging between enhancer and promoter and between silencers (Fig. 6A). Additionally, insulator proteins, such as dCTCF associated with cohesins, may contribute to the structural but also selective confinement of active/repressive modules by forming “insulated neighborhood” (Dowen et al. 2014) (Fig. 6A). At larger scale, TADs that can be either constitutively or facultatively formed during development contribute also to the confinement of the “sub-TADs” modules, providing a “basal” (large-scale) level of confinement and of selectivity that are then finer-tuned at lower scale within sub-TADs modules (Le Dily et al. 2014). Implication of TADs in regulating transcription has been also recently proposed in the process of mammalian X inactivation (Tiana et al. 2016; Giorgetti et al. 2014): consistently with this nanoreactor hypothesis, the expression of the Tsix transcript was positively correlated with the compaction level of its embedding TAD. And more generally, recent studies have shown that perturbing TAD integrity may indeed lead to transcriptional deregulation and diseases (Guo et al. 2015; Lupianez et al. 2015, Lupiáñez et al. 2016; Flavahan et al. 2016; Hnisz et al. 2016, Franke et al. 2016).

### The self-assembly of structural and functional compartments: the “living chromatin” framework

As pointed before, active and inactive structural domains are also often characterized by a well-defined epigenomic state. These local chromatin states are characterized by a distribution of specific chromatin marks that may favor the selective chromatin/DNA binding of regulatory proteins. These epigenomic marks are deposited and removed by specific enzymatic complexes (e.g., p300, PRC2, Su(Var)3–9, Sir2, etc.) that can associate with the mark they catalyze (H3/4KAc, H3K27me3, H3K9me2/3) and that are often associated with architectural proteins (TFs, PRC1, HP1, Sir3) that promote bridging between distant sites and thus compartmentalization. Such “reader-writer/eraser-bridger” mechanism enables the mark and thus the chromatin state to spread once nucleated at some specific genomic loci (Beisel and Paro 2011; Simon and Kingston 2013; Zhang et al. 2015; Chen and Dent 2014; Soshnev et al. 2016). The crucial point here is that spreading might operate not only *in cis* but also *in trans* to any chromatin fragment that are in the spatial vicinity. This would introduce a positive feedback between the local chromatin state dynamics and the global compaction level: within a given domain, the compaction would enhance the “spreading” of the chromatin state over the entire domain which in return would enhance global compaction (Fig. 6B). Some experiments have pointed out the possible role of “long-range” spreading *in trans* in maintaining epigenomic domains (Obersriebnig et al. 2016). In the dosage compensation mechanism, experiments suggest that the global compaction indeed may influence the establishment of the inactivated (in mammals), downregulated (in *Caenorhabditis elegans*) and upregulated (in *Drosophila*) states by long-range spreading of H3K27me3/PRC1, Ac, and DCC/H4K20me1 from initiation sites (Ferrari et al. 2014). In the mammalian system, some further evidence of the dynamic coupling between epigenome assembly and compaction has also been recently proposed (EngreitzJ et al. 2013). Similar structural coupling might also drive the formation of hyperacetylated mega-domains as recently observed in NUT midline carcinoma (Alekseyenko et al. 2015).

Theoretical investigations with quantitative prediction of how confinement and spatial folding may affect expression and epigenomic regulation have been mainly developed in the context of chromatin/DNA looping (Vilar and Leibler 2003; Doyle et al. 2014; Liu et al. 2016). However, they actually mainly focused on the effect of one single loop, and further modeling of enhancer/promoter communication in a chromosomal context such as in TADs or higher order compartments will be needed to better understand the functional role of such compartments. Recent works on the computational modeling of the epigenome assembly based on the reader-writer properties have shown how an efficient establishment and a robust maintenance of epigenomic domain indeed require long-range spreading (Angel et al. 2011; Dodd et al. 2007; Jost 2014); however, these models are essentially 1D models with an ad hoc introduction of confinement and with no feedback effect of histone marks/chromatin state on the level of confinement.

We, and other groups (Broederz et al. 2014; Michieletto et al. 2016), are currently working on the development of a proper theoretical framework that explicitly couples the spreading of marks and the folding of the chromatin. We propose to refer to such model as the living chromatin model which is basically a copolymer model as presented in the previous section but with the local monomer states that can switch between different states according to basic transition rules that will depend on the spatial folding of the chain (Fig. 6C). In a simplified version of such approach, the local epigenomic state *e*_*i*_ of monomer *i* is allowed to fluctuate between two states (−1 for repressive and +1 for active). The reader-writer mechanism (in *cis* and in *trans*) imposes that −1 (resp. +1) monomers “aim” to propagate their states to their 3D neighborhood. This is modeled by assuming that the dynamics of the epigenome is driven by the Ising-like Hamiltonian *H*_*epi*_ =  − (*J*_*e*_/*N*) ∑ *δ*_*i* , *j*_ *e*_*i*_ *e*_*j*_ with *J*_*e*_(>*0*) the strength of the epigenomic spreading and *δ*_*i* , *j*_ = 1 (resp.0) if monomers *i* and *j* are (resp. not) spatially in contact. The dynamics of the (co)polymer chain is driven by specific interactions that depend on the local epigenomic landscape ${H}_{inter}=-\sum {J}_i\left({e}_i{e}_j+1\right)/2\mathit{\exp}\left(-{r}_{ij}^2/\left(2{r}_o^2\right)\right)$ with *J*_*i*_(>*0*) the strength of interaction between monomers of the same epigenomic state. Coupling the GSC approach (including the crumpling effect) to a mean-field approximation for the epigenome dynamics allows to efficiently study the system for any parameters *J*_*e*_ and *J*_*i*_ (Fig. 6D). Within this framework, we ask if it is possible to establish and maintain a coherent epigenomic state in an insulated genomic region (*N* = 100), i.e., with almost all the monomers in the same *e*_*i*_ at the same time. For weak spreading, the reader-writer mechanism is not strong enough to maintain a stable coherent state. For a given *J*_*i*_, as *J*_*e*_ is increased, the system observes a phase transition and fluctuates stochastically between a coherent active or inactive state, the residence time in each macrostate increasing exponentially with *J*_*e*_ (Jost 2014). This illustrates that long-range spreading (via the 3D polymeric structure) between distant loci along the linear genome is a key factor to epigenome maintenance. Strikingly, the position of the critical point is a decreasing function of *J*_*i*_. This implies that introducing specific epigenomic-associated contact interactions allows the quick establishment and the strong stabilization of a coherent state at weaker, easier to control, spreading efficiency. Indeed, 3D concentration of reader-writer-bridger complexes is locally increased leading to enhanced cooperative effects.

In vivo, the situation is clearly more complex. Initiation (nucleation) for de novo activation/repression may be performed via the primary targeting of activating/silencing complexes to specific genomic sites mediated by DNA-binding proteins (or by non-coding RNAs) which is then followed by the coupled self-assembly of the chromatin and structural state (Noordermeer et al. 2011; Cheutin and Cavalli 2012) that further enhances activation/repression and perpetuates the active/repressive environment throughout cell division. This may correspond to a conversion from (i) a “hard-wired” targeting of regulators to few discrete recruitment and bridging genomic sites at the induction stage (in response to developmental or environmental cues), to (ii) a “soft-wired” targeting of regulators with weaker interactions but associated to a larger number of spatially concentrated secondary sites at the maintenance stage (no more or lower cues). In that context, the structural/functional nuclear compartments would correspond to the self-assembly of a robust nanoreactor where the biochemical reactions (DNA/chromatin binding of regulators, multimerization of regulators, spreading of marks, transcription, replication, etc.) would both depend on and induce (reinforce) their spatial confinement. Testing this hypothesis of (self-assembled) nanoreactor will require dedicated experimental and theoretical investigations. And, besides the need to further improve the modeling of structural properties of chromosomes at all scales, and in particular to develop dynamical model of spatial organization, we stress that it might be the time for physicists to engage in the quantitative modeling of the functional consequence of spatial compartmentalization.

## Abbreviations

GSC: Gaussian self-consistent
TAD: Topological associating domains
